# Reoperation Risk, Bone Health, and Alignment Targets in Proximal Junctional Kyphosis After Adult Spinal Deformity Surgery

**DOI:** 10.1016/j.xnsj.2026.100932

**Published:** 2026-07-17

**Authors:** Alexander Kucherina, Themistocles Protopsaltis

**Affiliations:** Department of Orthopedic Surgery, NYU Langone Orthopedic Research Institute, NYU Langone Health, 380 2nd Ave, 6th Floor, New York, NY 10003, United States

Ohba et al. recently published a retrospective cohort study evaluating how proximal junctional kyphosis (PJK) subtype and osteoporosis treatment influence reoperation risk following adult spinal deformity (ASD) surgery, examining whether PJK morphology and the timing of anabolic therapy, particularly teriparatide, could stratify mechanical failure risk and guide perioperative management [1].

This topic is clinically important because long-segment fusion for ASD carries substantial mechanical complication risk, and PJK and proximal junctional failure (PJF) can compromise sagittal alignment, impair quality of life, and frequently require revision surgery [[2], [3]]. Fewer studies have explored whether distinct PJK morphologies carry different prognostic implications or how bone health optimization may modify these risks.

The authors retrospectively reviewed 189 patients older than 60 years who underwent long-segment ASD fusion at a single institution between 2016 and 2023, classifying PJK by morphologic subtype (Type 1 soft tissue or ligamentous failure, Type 2 vertebral fracture, Type 3 implant or bone-implant interface failure) and angular severity, while also assessing bone mineral density (BMD), osteoporosis medication exposure, and timing of anabolic therapy. PJK developed in 95 patients (50.3% of the cohort), and reoperation risk varied markedly by subtype: Type 3 had the highest revision rate (69.2%), followed by Type 2 (38.1%), while Types 0 and 1 were substantially lower (2.1% and 6.1%, respectively). Increasing angular severity was also associated with higher revision rates, reinforcing that PJK is not a homogeneous complication; mild or ligamentous failure is often clinically manageable, whereas vertebral fracture and implant-related failure represent more aggressive structural phenotypes with substantially greater revision burden.

Lower BMD was associated with more severe PJK patterns, particularly Type 2, consistent with prior literature showing that osteoporotic bone quality increases the risk of screw loosening, vertebral fracture, and construct failure after ASD correction [[4], [5]]. Notably, patients treated with teriparatide demonstrated different distributions of PJK subtype and severity, including fewer Type 2 and severe Grade B/C deformities, and preoperative teriparatide was associated with numerically lower (though not statistically significant) reoperation rates, consistent with prior evidence that teriparatide improves vertebral bone microarchitecture and reduces bone-failure-type PJK [6], a benefit recently supported by prospective data on perioperative teriparatide in osteoporotic thoracolumbar deformity correction [7].

Strengths of this study include its clinically meaningful PJK classification, integration of bone health variables, and a relatively substantial single-center cohort, moving beyond a binary PJK definition toward a more nuanced framework for risk stratification, given that PJK severity has been shown to correlate with pain and outcomes [8]. However, the retrospective single-center design limits causal inference and introduces possible selection bias; teriparatide-treated patients may have differed from untreated patients in unmeasured ways, and medication adherence, duration, and timing were difficult to standardize. The lack of patient-reported outcomes prevents direct correlation of PJK subtype with pain or quality of life, and subgroup analyses of less frequently used medications were likely underpowered.

These findings should also be interpreted within the broader context of individualized sagittal alignment planning, since PJK is multifactorial and bone health is only 1 component of risk-based alignment overcorrection, construct rigidity, upper instrumented vertebra selection, paraspinal muscle quality, and soft tissue preservation all influence junctional biomechanics. Protopsaltis et al. demonstrated that alignment targets should vary by age and pelvic incidence rather than fixed, age-independent normative thresholds [9], and in a subsequent study found that correction toward normative alignment yielded lower postoperative TPA and PI-LL values but higher rates of PJK and PJF without corresponding quality-of-life benefit, compared with correction toward age-appropriate functional alignment [10]. Lafage et al. similarly showed that patients with PJK, particularly older patients, were more often overcorrected relative to age-adjusted alignment goals [11]. Together, these studies suggest that meaningful PJK prevention requires harmonizing biologic and mechanical risk factors, and Ohba et al. add that once PJK occurs, its morphology strongly influences revision risk.

Overall, this study advances risk stratification in ASD surgery by showing that PJK should not be treated as a uniform outcome: structural subtypes, especially vertebral fracture and implant-related failure, carry far higher reoperation risk than mild or ligamentous forms, and the teriparatide findings support incorporating preoperative bone health assessment into surgical planning. Future prospective multicenter studies should evaluate standardized osteoporosis protocols, patient-reported outcomes, and alignment targets stratified by age, pelvic incidence, and functional status, ultimately balancing undercorrection-related disability against overcorrection-related junctional failure.
